# Clinical and Radiological Profiles of COVID-19 Patients with Neurological Symptomatology: A Comparative Study

**DOI:** 10.3390/v13050845

**Published:** 2021-05-06

**Authors:** Maria de Fatima Viana Vasco Aragao, Mariana de Carvalho Leal, Pedro Henrique Pereira Andrade, Ocelio Queiroga Cartaxo Filho, Lucas Vasco Aragao, Tatiana Moreira Fonseca, Marcelo Andrade Valenca, Maria Regina Vendas Carneiro Leao, Joao Pedro Vasco Aragao, Maria Lúcia Soares, Mirelle Palmeira Lima, Silvio S. Caldas, Marcelo Moraes Valenca

**Affiliations:** 1 Centro de Biociências, Anatomy Department, Universidade Federal de Pernambuco, Recife 50670-901, Brazil; 2 Centro Diagnostico Multimagem, Recife 50070-450, Brazil; 3 Surgery Department, Post Graduation on Helth Human Communation, Universidade Federal de Pernambuco, Recife 50670-901, Brazil; marianacleal@hotmail.com (M.d.C.L.); oceliocartaxo@terra.com.br (O.Q.C.F.); tatimoreiraf@gmail.com (T.M.F.); silviodasilvacaldasneto@gmail.com (S.S.C.); 4 Real Hospital Português de Beneficência em Pernambuco, Recife 50050-290, Brazil; mirellepll@gmail.com; 5 Centro de Ciências Médicas, Faculdade de Medicina do Recife, Universidade Federal de Pernambuco, Recife 50670-901, Brazil; pedroandrade.phpa@gmail.com (P.H.P.A.); marcelo.a.valenca@gmail.com (M.A.V.); mreginavcleao@gmail.com (M.R.V.C.L.); 6 Endoscopy Department, Hospital 9 de Julho, São Paulo 01409-002, Brazil; lvaragao13@gmail.com; 7 Faculdade de Medicina de Olinda, Olinda 53030-030, Brazil; joaopedro_aragao@hotmail.com; 8 Propedeutics Department, Áreas de Ciências da Saúde, Faculdade de Medicina, Universidade Federal de Alagoas, Alagoas 57072-970, Brazil; glmourao@icloud.com; 9 Department of Morphological and Functional Organization 1, 2 and 3, Centro Universitário Cesmac, Alagoas 57051-160, Brazil; 10 Centro de Ciências Médicas, Surgery Department, Universidade Federal de Pernambuco, Recife 50670-901, Brazil; 11 Centro de Ciências Médicas, Pós Graduation Neuropsquiatry Department, Universidade Federal de Pernambuco, Recife 50670-901, Brazil; mmvalenca@yahoo.com.br

**Keywords:** COVID-19, MRI, CT, brain, stroke, olfactory bulb, radiology, risk factors

## Abstract

Patients with COVID-19 can require radiological examination, with chest CT being more frequent than neuro-imaging. The objective is to identify epidemiological, clinical and radiological factors considered as predictors of neurological involvement in patients with COVID-19 assessed by neuroimaging and to describe the neuroimaging findings. This retrospective study was performed with 232 consecutive confirmed COVID-19 patients, from two radiological units, which were divided into two groups: (1) those who underwent a brain CT/MRI scan (n = 35) versus (2) those who did not undergo the brain CT/MRI scan, but underwent only chest CT (*n* = 197). There was a statistically significant difference with associations regarding the COVID-19 brain scan group for: admission to ICU, greater severity of lung injuries, the use of a mechanical ventilator and sepsis. Statistical tendency was found for chronic renal failure and systemic arterial hypertension. Forty-percent of COVID-19 patients from the brain scan group were abnormal on brain CT and/or brain MRI (22.9% of the cases with bleeding or microbleeding, 8.6% with restricted diffusion lesions). One ischemic stroke case was associated with irregularity at the M1 segment of the right middle cerebral artery. There was a case of left facial nerve palsy with enhancement of the left geniculate ganglia. An analysis of the olfactory bulbs was possible in 12 brain MRIs and 100% had enhancement and/or microbleeding. In conclusion, a more severe COVID-19 disease from ICU, a more severe form of lung disease, the use of mechanical ventilator and sepsis were associated to the COVID-19 patients with neurological involvement who had undergone brain scans. Microvascular phenomenon was a frequent finding in the brain and olfactory bulbs evaluated by neuroimaging.

## 1. Introduction

Different abnormalities in both peripherical and central nervous systems were reported in patients with COVID-19, such as vessel wall enhancement and/or focal cerebral arteriopathy; Reference [1] acute ischemic infarct; References [1,2] hemorrhage; References [2,3] acute hemorrhagic necrotizing encephalopathy; Reference [4] cerebral venous thrombosis; References [5,6] diffuse leukoencephalopathy [7] with microhemorrhage; References [8,9] PRES-like with microvessel enhancement; Reference [10] splenium of corpus callosum restricted diffusion lesion; Reference [11] edema [12,13] and enhancement and microbleeds of olfactory bulbs; and Reference [14] as well as bilateral facial palsy and enhancement of facial nerves [15].

There is a spectrum of severity in the clinical presentation of patients with COVID-19, from asymptomatic individuals to very severe comatose patients with ventilatory failure.

For this reason, COVID-19 patients are assessed differently, depending on the symptoms or signs seen in the course of the disease. This can indicate, or not, the need of further investigation by laboratorial and radiological exams, such as computed tomography of the chest or an MRI of the brain.

It is observed that a small number of all patients infected with SARCoV2 need to have a further investigation by these radiological exams. However, these patients in the pandemic context are saturating the health system, especially the hospitals and radiology departments. It is not fully understood why some have the worst evolution with only lung failure and others also with brain injury.

The identification of clinical, epidemiological factor and those groups of higher risk that may be more related to neurological manifestation in patients with COVID-19 can help in the prevention of complications and in early diagnosis of appropriate treatment, in an effort to minimize the sequelae and fatal cases, thus improving the prognosis of patients.

Based on the principle that patients with neurological complaints and possible intracranial lesions as attributable causes, evaluated with brain magnetic resonance imaging (MRI) or brain computed tomography (CT), we decided to assess two groups of patients comparatively: (1) a group of patients with COVID-19 assessed by neuroimaging compared to (2) a group of COVID-19 without any brain imaging evaluation looking for differences that may indicate associated factors with neurological impairment.

The purpose of this study is to identify epidemiological, clinical and radiological factors considered as predictors of neurological involvement in patients with COVID-19 assessed by neuroimaging, and also to describe the neuroimaging findings.

## 2. Method

The Institutional Review Board of the Ethics Committee of the “Universidade Federal de Pernambuco” approved this retrospective study. Informed consent was waived.

A survey of the cases was carried out to identify the patients with confirmed COVID-19 who underwent chest CT scan and/or brain MRI/CT in two radiology departments.

The radiological and clinical data were retrospectively collected from 28 March 2020 to 3 September 2020. The origin of the data was from two radiological units in Recife, Pernambuco, Brazil: “Multimagem Centro Diagnóstico” and “Real Hospital Português de Beneficência em Pernambuco”.

**Figure 1:**
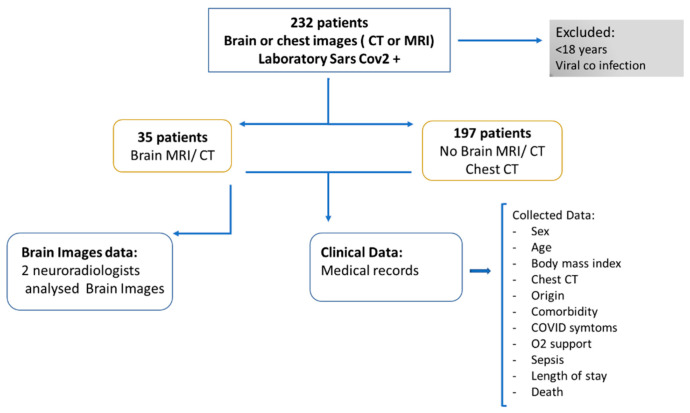
Block Diagram of the proposed research.

As is shown in the block diagram of the proposed research (Figure 1), we used a convenience sample of 232 cases, for which we were able to collect the clinical data from medical records in time to analyze and write this study which: (a) had laboratory confirmation of COVID-19, (b) excluded patients under 18 years of age and with co-infection of another viral infection.

The 232 confirmed COVID-19 patients were divided into two groups: (1) those who underwent a brain CT/MRI scan (*n* = 35) and (2) those who did not undergo a brain CT/MRI scan, but underwent a chest CT (*n* = 197).

**Figure 2:**
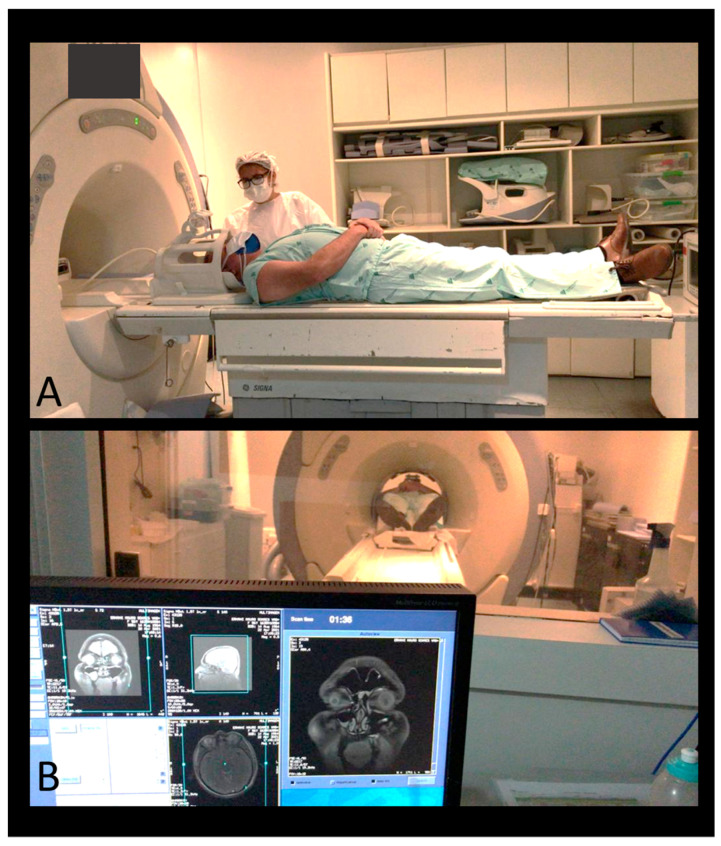
Moments during the performance of MRI on the brain of a patient with COVID-19 (**A**) Patient being positioned in the MRI machine with the brain coil having been placed. (**B**) Photograph of the MRI Control Room during the examination and the acquisition of the brain magnetic resonance images of a patient with COVID-19.

**Figure 3:**
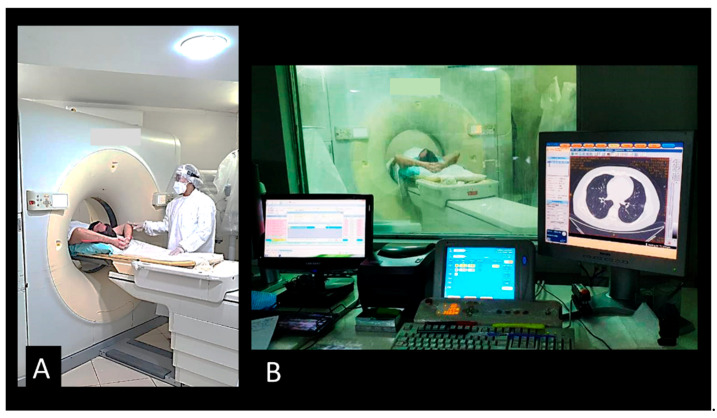
Moments during the performance of a chest computerized tomography of a COVID-19 patient. (**A**) Patient being positioned in the computerized tomography machine for chest examination without contrast. (**B**) Photograph of the Computerized Tomography Machine Control Room during a computerized tomography examination of the chest of this patient.

Figure 2 shows the magnetic resonance and Figure 3 shows the computerized tomography machines during the exams of two COVID-19 patients who are not of this sample to illustrate this article.

The Institution’s radiologists initially analyzed all chest and brain scans. Atypical pneumonia CT patterns for COVID-19 were excluded. The lung opacities qualitative scoring of COVID-19 (virus pneumonia) patterns was performed independently by the Institution’s radiologists: (a) less than 25%, (b) between 25% and 50% or (c) equal to or more than 50%. Our study only considered the evaluation of the Institution´s radiologists of chest CT reports.

Subsequently, all images of brain CT and MRI were reviewed independently by two radiologists and neuroradiologists who were certified by the Ministry of Education and Culture and Brazilian College of Radiology with 30 (M.F.V.V.A.) and 18 years (O.Q.C.F.) of experience, respectively. In cases where there was disagreement, the results were resolved by consensus.

The clinical variables data, retrospectively collected from the medical records, were: (a) sex; (b) age; (c) the percentual extent of pulmonary lesions found in the chest CT; laboratory SARS-CoV2 infection confirmation; abnormalities found in brain CT or MRI, (d) presence of cough, headache, anosmia, ageusia, dyspnoea, systemic arterial hypertension, dyslipidemia, obesity, renal failure, use of mechanical ventilation, and a neurological complaint. The origins of patients were also assessed, whether they were admitted to the Emergency Room, hospitalized or admitted to the ICU. It was also assessed whether a patient had progressed to death before the assessment date.

An analysis of the olfactory bulbs was possible in 12 brain MRIs because the 12 had at least a sequence of coronal thin slices pre- and/or post-contrast fat suppressed T1WI and one patient had pre- and post-contrast 3D SPGR T1WI and also 3D FLAIR.

The intensity of olfactory bulbs was defined as normal when the bulbs had the same cortex intensity, as typically seen in healthy controls. Abnormal olfactory bulb intensity is when the bulbs are more hyperintense than the cortex on T1WI and STIR.

After gadolinium injection on T1WI, enhancement of the olfactory bulbs is defined when they become more hyperintense, in comparison to their intensity on pre-gadolinium T1WI. However, when there is only the post-gadolinium T1WI and the bulb is more hyperintense than the normal cortex, this represents olfactory bulb intensity abnormality and maybe an enhancement or microbleeding (methaemoglobin), as interpreted in the present study. Microbleeding (methaemoglobin) in the olfactory bulb is considered when there is hyperintense olfactory bulb, compared to the normal cortex or the normal contralateral bulb, on pre-gadolinium fat suppression TIWI.

### Statistical Analysis

Softwares SPSS 13.0 (Statistical Package for the Social Sciences) for Windows and Excel 2010 were used; all tests were applied with a 95% confidence.

The results are presented in table form with their respective absolute and relative frequencies. The numerical variables are represented by measures of central tendency and measures of dispersion.

To verify the existence of an association: Chi-square Test and Fisher’s Exact Test for categorical variables were applied.

## 3. Results

Of 232 confirmed COVID-19 patients, 35 had undergone brain scans. Thirty of these 35 had chest CT performed as well.

Table 1 shows the profile of a group of 35 COVID-19 patients that underwent brain imaging scans, compared to a group of 197 cases without brain scans (but underwent chest CT).

There were associations regarding the COVID-19 group, which underwent brain-imaging scans for the origin within the hospital (more frequent in ICU patients), greater severity of lung injuries, the use of mechanic ventilators, complaints of dyspnea (less frequent), and sepsis. There was a statistical tendency for chronic kidney failure and systemic arterial hypertension.

**Figure 4:**
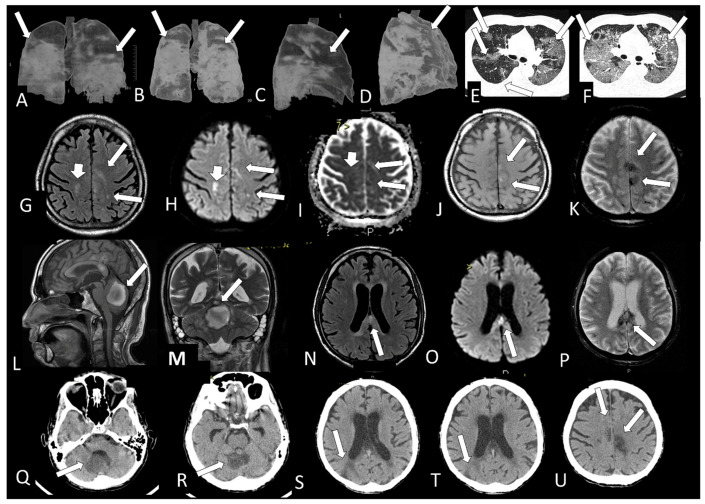
Case 31—The Chest CT on admission shows areas of alveoellar consolidation and ground glass in more than 50% of lung area (arrows **A**,**C**,**E**). Three days later, there was a severe increase of the lung disease (arrows, **B**,**D**,**F**). The brain MRI shows some hyperintense small areas on FLAIR (arrows, **G**) and in DWI (arrows, **H**) which are hypointense on ADC-Map (restricted diffusion lesions, arrows, **I**) in both cerebral hemispheres. Small areas of microbleeding are observed, being hyperintense on T1WI (**J**, arrows) and hypointense on T2* (**K**, arrows) localized on the medial part of left pre and postcentral gyrus. There is also a large oval hematoma located at the vermis of the cerebellum, being hyperintense on sagittal T1 (**L**, arrow) and on coronal T2WI (**M**, arrow) with peripheric hypointense ring of hemosiderin. Some small lesions, which represent microbleeding (methemoglobin), are also located on the splenium of the corpus callosum hyperintense on FLAIR (**N**, arrow) and on DWI (**O**, arrow) being hypointense on T2* (**P**, arrow). The CT (**Q**–**U**) without contrast made one month later showed that the lesions described above on previous MRI are hypointense and that a new sequel in the right parietal lobe (**S**,**T**, arrow) had appeared.

**Figure 5:**
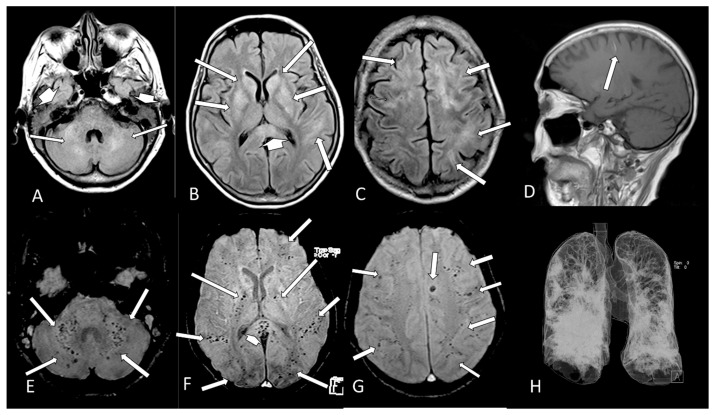
Case 30—The brain MRI shows multiple and confluent areas of diffuse hyperintensity on FLAIR (**A**–**C**, arrows) and liquid in the mastoid cells (**A**, short head of arrows). A small linear hyperintense subcortical on sagittal T1 (**D**, arrow), which represents methemoglobin, is observed. There are multiple small dots of micro bleeds at the cerebellum and middle cerebellar peduncle (**E**, arrows), intern capsule (**F**, long arrows), splenium of corpus callosum (**F**, head of arrow) and subcortical white matter (**F**,**G**, arrows) of the brain. The 3D chest CT reconstruction shows that there is more than 50% of opacification in both lungs (**H**).

**Figure 6:**
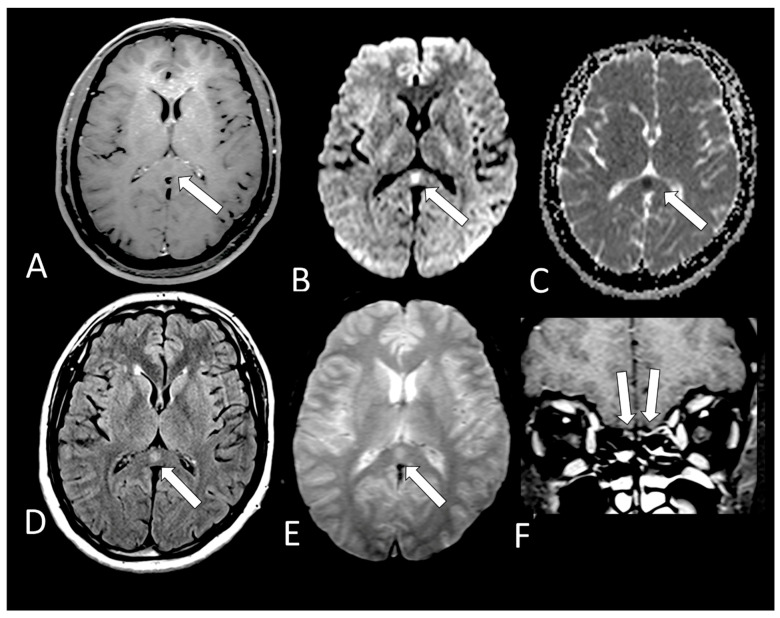
Case 18—On MRI, there is a small round lesion on the splenium of the corpus callosum which could represent a cytotoxic lesion due to a cytokine storm and differential diagnosis is with small acute infarct. It is hypointense on T1 (**A**, arrow) with restricted diffusion, being hyperintense on DWI (**B**, arrow) and hypointense on ADC-Map (**C**, arrow). This lesion is also hyperintense on FLAIR (**D**, arrow) and T2* (**E**, arrow) without microbleeding. The olfactory bulbs are hyperintense on coronal post contrast fat suppressedT1WI (**F**, arrows) in comparison to the gray matter of the frontal lobes, which can represent enhancement, but we cannot exclude microbleeding.

**Figure 7:**
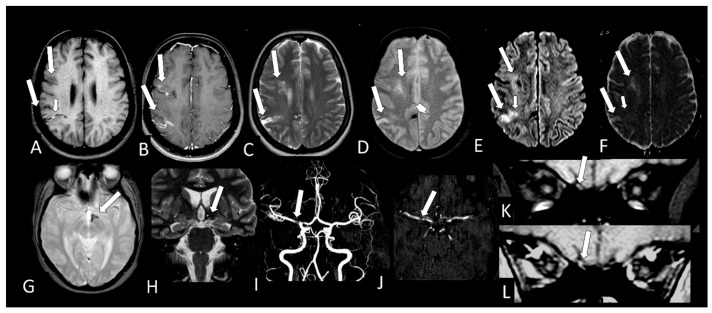
Case 34—The MRI shows a predominantly chronic ischemic stroke, located in the cortical and subcortical region of the right middle cerebral artery territory (M8 and M10) (**A**–**F**, long arrows). In the right M10 territory, the lesion has a small hyperintense “line” on T1 (**A**, short arrow) probably methemoglobin; the cortical enhances with contrast (**B**, long arrows); is hyperintense on T2 and T2*(**C**,**D**, long, arrow), being hyperintense in DWI (**E**, long arrows) and hyperintense on ADC-Map (**F**, long arrows), representing facilitates diffusion (T2 shine through effect). However, there is still a small area in M10 lesions that shows restricted diffusion of probably more recent ischemic injury being hyperintense in DWI (**E**, small arrow) and hypointense in ADC-Map (**F**, small arrow). There are small areas of bleeding with magnetic susceptibility in T2 * (**D**, arrow head) located in the medial cortical of the right cerebral hemisphere and in the left wall of hypothalamus (**G**, arrow) which is reduced in volume on coronal T2WI (**H**, arrow). On the angio MRI, parietal irregularity and narrowing in the right M1 is observed (**I**,**J**, arrow) and also in the origin of right anterior artery. Small dot of hyperintensity in the right olfactory bulb (**K**, arrow) observed on pre contrast fat suppressed T1WI, which seems have increased signal with contrast (**L**, arrow) representing probably methemoglobin and enhancement.

**Figure 8:**
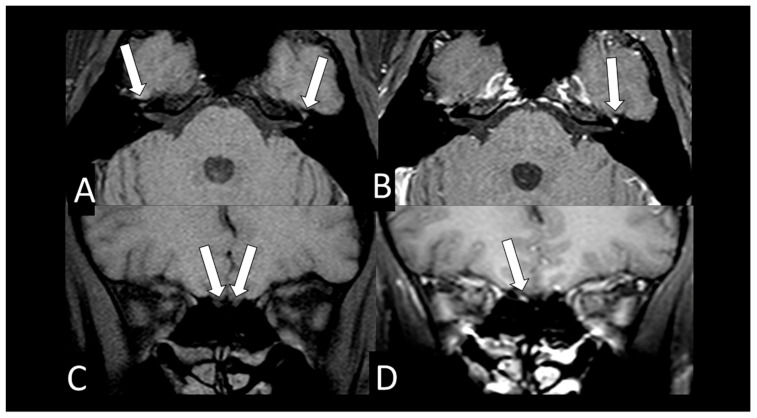
Case 32—The MRI shows both hypointense geniculate ganglions on axial fat suppressed T1 WI (**A**, arrows), but the left geniculate ganglion has abnormal stronger enhancement on axial post contrast (**B**, arrow) fat suppressed T1 WI. The coronal fat suppressed T1 WI shows olfactory bulbs with normal hypointense signal (**C**, arrows), but the post contrast coronal fat suppressed T1WI shows abnormal enhancement in the right olfactory bulb (**D**, arrow).

**Figure 9:**
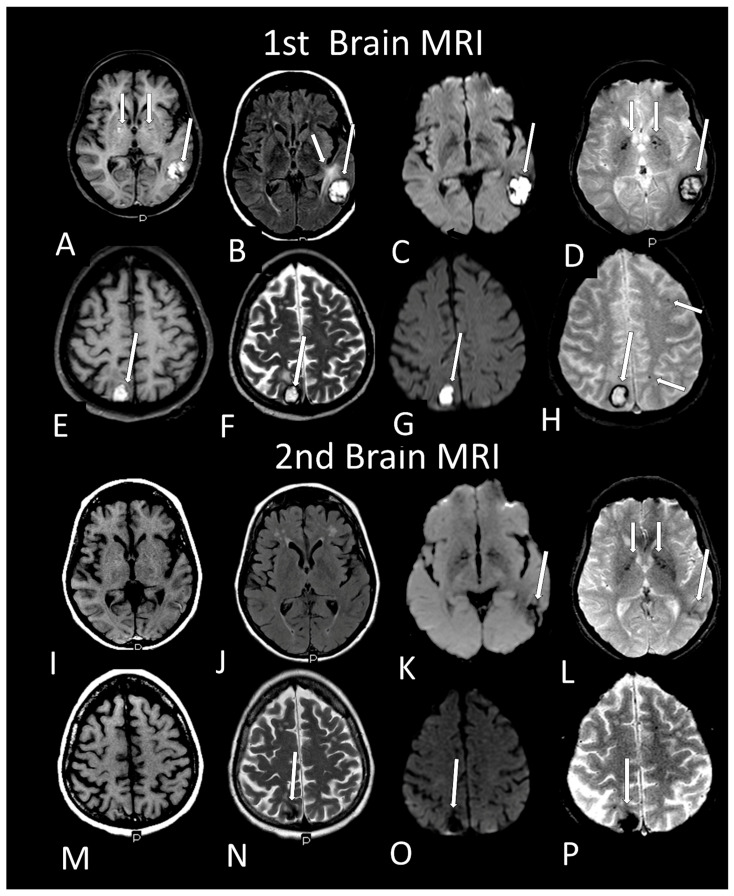
Case 27—On MRI, there is a subcortical hematoma located respectively in transition between left posterior temporal lobe and occipital lobe (long arrow **A**–**D**) with hyperintense methemoglobin inside on T1WI (**A**, long arrow), FLAIR (**B**, long arrow), DWI (**C**, long arrow) and T2* (**D**, long arrow) and with hemosiderin in the periphery being hypointense ring. There is vasogenic edema near the left temporal hematoma being hyperintense on FLAIR (**B**, short arrow). There is also microbleeding in pale globes (**A**,**D**, short arrows). Another cortical hematoma in the right parietal lobe (**E**–**H**, long arrow), with the same characteristics described above with methemoglobin inside and hemosiderin in the periphery. There are small dots of subcortical microbleeding in the left frontal and parietal lobes (**H**, short arrows). Two months later there was regression on MRI (**I**–**P**) of both hematomas in size, but deposition of hemosiderin remained being hypointense on all sequences [T1, (**I** and **M**); FLAIR, (**J**); DWI, (**K**,**O**, arrow); T2, (**N**, arrow); and T2* (**L**,**P** arrows)] and in the lentiform nuclei (**L**, small arrows).

**Figure 10:**
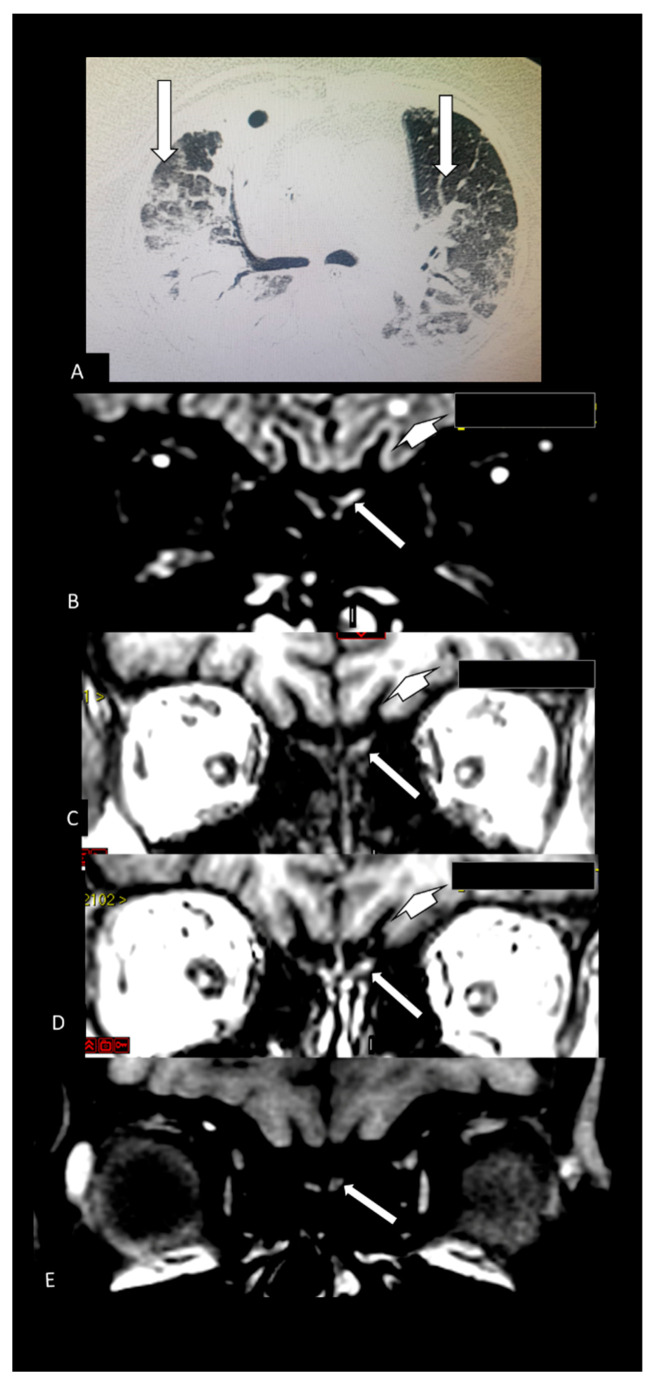
Case 27—The Chest CT shows more than 50% of typical lung opacity of COVID 19 pulmonary injury, bilaterally (**A**, arrows). The MRI shows hyperintense lesion on 3D FLAIR (**B**, arrow) [16] and also on pre (**C**, arrow) [16] and post contrast SPGR T1 WI (**D**, arrow) [16]. This is suggestive of component of probably methemoglobin in this left olfactory bulb lesion which seems be little bigger and asymmetric compared with the apparently normal right olfactory bulb. This asymmetry in size is better seen on FLAIR (**B**) [16]. There is also a small round hyperintense lesion in the subcortical white matter in the left frontal lobe on FLAIR (**B**, short arrow) which is hypointense on T1WI (**C**, short arrow) and does not enhance on post-contrast T1WI (**D**, short arrow) being nonspecific [16]. The MRI performed 3 months later shows on pre contrast fat suppression T1 WI (**E**) that there is reduction on hyperintensity of the left olfactory bulb lesion.

Table 2 shows the individual data of demographic, clinical and radiological characteristics of the 35 adult patients with COVID-19 on whom brain MRI and/or CT scans were performed because of clinical neurological complications. Among those 35 patients, the brain CT and/or brain MRI were considered normal in 21 (60.0%) of them. Abnormalities were identified in 14 (40% of the patients). Regarding the frequency of abnormality (Figure 4, Figure 5, Figure 6, Figure 7, Figure 8, Figure 9 and Figure 10) among the 35 patients with neuroimaging investigation, we observed the following: (a) 8/35 (22.9%) cases with bleeding or microbleeding (Figure 4K–M,P; Figure 5E–G; Figure 7A,D,G and Figure 9); (b) 3/35 (8.6%) cases with restricted diffusion lesions being all illustrated here (one case with restricted DWI small areas had also associated multiple small areas of microbleeding (Figure 4H,I)); one case had restricted diffusion lesion only in the splenium of the corpus callosum without any microbleeding in the brain (Figure 6A–E), and with olfactory bulbs injury (Figure 6F); and one case of ischemic stroke involving the right middle cerebral artery territory (Figure 7A,B, arrows) with irregularity at the right middle cerebral artery (Figure 7I,J, arrows) and olfactory bulb injury (Figure 7K,L, arrows)]; (c) 2/35 (5.7%) cases with previous old stroke lesion (no relation with Covid-19); (d) 1/35 (2,8%) case with left facial nerve palsy and enhancement of geniculate ganglia (Figure 8A,B) and right olfactory bulb/tract injury with post contrast enhancement (Figure 8C,D).

All 12 brain MRIs (100%), where it was possible to evaluate the olfactory bulbs (Table 2), had injury in the olfactory bulbs, suggestive of enhancement and/or methehemoglobine (Figure 6F; Figure 7K,L, Figure 8C,D and Figure 10B,D). Six of them (50%) had normal brain MRIs and the other six (50%) showed brain MRI abnormalities as well (Table 2).

It was only possible to evaluate the olfactory bulbs in only 12 brain MRIs (Table 2). In all of them, injury in the olfactory bulbs, suggestive of enhancement and/or methahemoglobine, was found (Figure 6F, Figure 7K,L, Figure 8C,D and Figure 10B,D). Six of the 12 (50%) had normal brain MRI. The abnormalities encountered in 6/12 (50%) are shown in Table 2.

## 4. Discussion

The profile of the COVID-19 patients’ group which needed to undergo brain CT and/or MRI was associated with more severe disease and admission to ICU, severe lung disease, use of mechanical ventilator and sepsis. They also had a statistical tendency for association with chronic renal failure and systemic arterial hypertension.

The knowledge of these factors can help clinicians to better monitor these patients an rate highly signs and symptoms that, when investigated, can lead to early diagnosis and treatment of neurological disorders, especially hemorrhagic events, the most frequent finding in our study (Table 2), which have morbidity and mortality directly related to the identification and decision-making as promptly as possible.

Despite the COVID-19 patients’ group which needed to undergo brain CT and/or MRI having severe lung disease and using mechanical ventilator, the complaint of dyspnea was statistically less frequent. However, this should be evaluated with caution because dyspnea complaint information was retrieved retrospectively from the medical record, and there may be underreporting.

When anosmia was being analyzed in our study, we did not find statistical differences when comparing the radiological group with brain scans to the radiological group without brain scans (but with chest CT). Another explanation may be because a bias arising from this information, which was only presented in 33.2% of the medical records.

Regarding brain imaging findings (without considering olfactory bulbs injury), 40% of the patients had abnormal imaging scans and all of them had vascular brain lesions. Brain haemorrhage lesions (bleeding or microbleeding) were a more frequent finding in our study and all the patients were ≥60 years old. The second main neuroradiological finding was the presence of lesions showing restricted diffusion, which can represent new ischemic lesions.

As in previous studies, the most frequent neuroimaging finding was single or multiple T2* punctiform hypointense lesions (microbleeding) [8,17,18,19,20] (frequently associated with white matter lesions on T2 [8,20], which sometimes have restricted diffusion) [8] located mainly at the subcortical white matter junction and sometimes at the splenium of the corpus callosum [17,18,19,20].

Several mechanisms for the SARS-CoV-2-related neurological complications are being considered: (a) a direct viral invasion (haematogenic or retrograde axonal by olfactory mucosa to olfactory bulbs) to the brain, which can lead to intracellular virus accumulation in endothelial cells (endothelitis with thrombotic microangiopathy) [17,18,19,20,21], neurons, Reference [22] glial cells, macrophages; (b) an indirect process resulting from hypercoagulability-related [23]; (c) an exaggerated cytokine [20]/immune-mediated response to viral infection causing damage to blood vessel wall or cells in the brain [24] with (d) ischemia, hypoxic, References [17,24] and (e) treatment complications [25] or (f) a combination of some or all of them [17].

We had one case in our study of splenium of corpus callosum restricted diffusion lesion without microbleeding. This finding was also described in previous studies [11,26,27], probably secondary to a cytotoxic lesion due to a cytokine storm, but even so, the possibility of acute ischemic injury cannot be ruled out.

The Wuhan study found that severe nervous system disease manifestations were more common in severe infections according to the American Thoracic Society guidelines for community-acquired pneumonia compared to nonsevere infections (45.5% vs. 30.2%) [28].Our study found almost the same information in relation to greater pulmonary extension of injury on chest CT, which was significantly associated with a radiological group of patients on whom brain scans had been performed because of neurological complication.

The primary role of the positive renin angiotensin system is to increase the sympathetic nervous system tension, cause vasoconstriction, increase blood pressure, and promote inflammation, fibrosis, and myocardial hypertrophy [29]. As an organ protector, the whole negative regulatory axis mediated by angiotensin couvert enzyme 2 (ACE2) can antagonize these effects [29]. However, the ACE2 is known to be a cell receptor for SARS-CoV [29] and now confirmed for SARS-Cov2 [29].

A study demonstrates that the ACE2 expression is increased in ischemic brains and vessels of patients with diabetes melitus and exposed to smoking, making them vulnerable to COVID-19 infection [30]. However, in our study, there was 25% of the patients with diabetes melitus and we did not find any differences between the two radiological groups studied. On the other hand, as a statistical tendency, systemic arterial hypertension was more frequent in the group submitted to neuroimaging investigation (52.9% vs. 22.0%; *p* = 0.062).

It is worth noting that in COVID-19 the “flu-like syndrome” and mild neurological symptoms [31] (e.g., headache) are very common, Reference [32] but that these symptoms are usually transient, disappearing in around half of the cases within 15 days [13,33,34,35,36], and for this reason, brain imaging (CT or MRI) is not regularly indicated for diagnostic clarification.

COVID-19 patients have presented symptoms of smell dysfunction that is frequently reported in various studies, from 5.1% to 88% of patients [13,28,33,35,37,37].

It was also described that the occurrence of anosmia or hyposmia has lower frequency (16%) for other viruses (as for example with influenza) when compared to COVID-19 [32]. Thus, there are clinical differences that can help the clinicians during the co-circulation of influenza and SARS-CoV-2 [32]. Due to this, anosmia is now considered as a criterion for testing for COVID-19 [38].

We only found 12 patients with COVID-19 with coronal thin slices on their brain MRI, thus making it possible to study the olfactory bulbs. All these patients (12/12) had anormalities in olfactory bulbs, suggesting microvascular phenomenon, such as microbleeding and/or enhancement by blood-brain barrier breakdown. Of those who had an olfactory bulb injury, 33.3% reported anosmia, 8.3% denied anosmia, and in the remaining 58.3%, there was no note in the hospital’s Records/Radiology Department about this complaint.

If we have evidence that there are lesions in the olfactory bulbs in patients with COVID-19, the anosmia/hyposmia symptom should be considered to be a frequent central neurological symptom and not only a flu-like symptom due only to involvement of the olfactory mucosa.

Despite these 12 cases (100%) with olfactory bulb injury having been investigated for a major neurological complication and not for anosmia, only six (50%) had other brain lesions not related to the olfactory bulbs. This finding can be the MRI documentation of which the olfactory bulbs injury has been related to the entry of the SARS-CoV-2 from the olfactory mucosa through the cribriform plate into the skull [14].

The natural history of SARS-CoV-2-induced anosmia has not yet been fully understood. Perhaps the damage to the olfactory epithelium (more related to an inflammatory process) or indirect damage to the neural cells would probably explain the cases of evolution with complete recovery. However, there are patients who evolve partial late recovery or even have not yet recovered.

Our study also suggests and supports the hypothesis that vascular phenomenon could be the main mechanism of COVID-19 in the brain and in the olfactory bulbs injuries as our previous scientific publications [14,16] and may also be the main mechanism of anosmia. Therefore, anosmia should be considered to be a neurological symptom and not just part of the flu syndrome in COVID-19.

Another interesting finding is that, despite mortality not being statistically different when comparing the two groups, it was slightly higher among patients investigated by brain neuroimaging (9.1% vs. 5.2%).

Fajnzylber and collaborators [39] found that the higher prevalence of detectable SARS-CoV-2 plasma viral load was associated to the greater the likelihood of developing the most severe forms of COVD-19 (with worse respiratory disease severity). The SARS-CoV-2 viral loads (specially plasma viremia) are also associated with increased risk of mortality. Unfortunately, in Brazil, the SARS-CoV-2 viral load assessment is not available in clinical practice. As our study is retrospective, information was collected from patients’ medical records without this information.

Thus, as a future perspective, we see the importance of deepening research mainly in the groups of greatest risk to developing the most serious forms of COVID-19. The groups of greatest risk should be studied in depth to find out why, and thus try to control (a) this factor, which increases the likelihood of complications in these groups, and (b) probably allow for major multiplication of the virus in these patients. In the future, science will be able to completely understand the pathophysiology of COVID-19 severe forms and develop some specific medication that can block the ease of SARS-CoV-2 viral action and multiplication of the virus in the patients in the high-risk groups.

The limitation of our study is that it is a retrospective one. The variables analyzed were not always noted in the medical records of all patients. Patients underwent brain scans because of major neurological complications and not on account of anosmia. For this reason, the MRI of most patients did not have an adequate sequence to evaluate the olfactory bulbs. Despite the anterior fossa region being analyzed with coronal fat suppression, T1WI can have susceptibility artifacts between the interface with the air, these artifacts are generally well recognized by radiologists and did not hinder the investigation nor the analysis [14,16,40,41].

In conclusion, more severe COVID-19 disease from ICU, a more severe form of lung disease, use of mechanical ventilator and sepsis were associated with the COVID-19 patients with neurological involvement who underwent brain scans. There was a statistical tendency to chronic renal failure and systemic arterial hypertension. Regarding brain imaging findings, 40% of the patients with neuroimaging investigation had abnormal imaging scans with all of them showing a vascular brain injury lesion, being more frequently microbleeding or bleeding, followed by restricted diffusion lesions. All the olfactory bulbs evaluated showed injury by microvascular phenomenon, probably methahemoglobine by microbleeding or microthrombus and/or abnormal enhancement by blood–brain barrier breakdown. Only one patient showed the left facial nerve palsy with enhancement of geniculate ganglia.

Prospective studies will be needed to better assess the risk groups, as well as the vascular neurological phenomena found, especially haemorrhagic ones and to relate them to viral load. This information is extremely important for the development of an adequate management of the treatment of severe forms of COVID-19 through adequate monitoring, early diagnosis and intervention, aiming to reduce morbidity and mortality. It can also contribute to the possibility of developing specific treatments for this terrible disease.

## Figures and Tables

**Table 1:** Comparison of the epidemiological, clinical and radiological factors between the COVID-19 patient groups that underwent CT and/or MRI of the brain and a control group that did not.

Variables	CT and/or MRI of the Brain		*p*-Value
	Yes	No	
	*n* (%)	*n* (%)	
Sex (Masculine)	20/35 (57.1)	115/197 (58.4)	0.892 *
Chest CT (Extension of pulmonary involvement)			
Normal	3/31 (9.7)	0/197 (0.0)	<0.001 **
Typical (T) < 25%	13/31 (41.9)	121/197 (61.4)	
T beween 25–50%	5/31 (16.1)	61/197 (31.0)	
T > 50%	9/31 (29.0)	14/197 (7.1)	
Between 25–50% with pleural effusion	0/31 (0.0)	1/197 (0.5)	
>50% with pleural effusion	1/31 (3.2)	0/197 (0.0)	
Origin			
Internal	14/33 (42.4)	103/197 (52.3)	<0.001 **
Intensive care unit	11/33 (33.3)	5/197 (2.5)	
Urgency	4/33 (12.1)	64/197 (32.5)	
External	4/33 (12.1)	25/197 (12.7)	
Systemic arterial hypertension	18/34 (52.9)	67/186 (36.0)	0.062 *
Diabetes melitus	10/32 (31.3)	44/184 (23.9)	0.376 *
Dyslipidemia	2/27 (7.4)	17/185 (9.2)	1.000 **
Asthma/COPD	2/29 (6.9)	25/173 (14.5)	0.382 **
Chronic kidney failure	4/30 (13.3)	7/182 (3.8)	0.053 **
Fever	24/29 (82.8)	151/188 (80.3)	0.757 *
Cough	22/29 (75.9)	159/189 (84.1)	0.289 **
Dyspnea	18/30 (60.0)	151/190 (79.5)	0.019 *
O_2_ catheter	14/29 (48.3)	78/189 (41.3)	0.477 *
Mechanic ventilatory assistance	10/29 (34.5)	19/190 (10.0)	0.001 **
Headache	12/28 (42.9)	78/188 (41.5)	0.891 *
Anosmia	7/11 (63.6)	50/66 (75.8)	0.462 **
Disgeusia	5/9 (55.6)	32/43 (74.4)	0.419 **
Gastric symptoms	7/26 (26.9)	61/190 (32.1)	0.594 *
Sepsis	7/9 (24.1)	12/189 (6.3)	0.006 **
Obesity (qualitative)	5/29 (17.2)	20/186 (10.8)	0.347 **
Death	3/33 (9.1)	10/192 (5.2)	0.413 **
Obesity (BMI ≥ 30)	2/9 (22.2)	35/78 (44.9)	0.289 **
	Mean ± DP	Mean ± DP	
Age (years)	54.3 ± 18.1	49.6 ± 13.7	0.163 ***
Body mass index	27.1 ± 6.3	29.6 ± 5.2	0.179 ***
	Median (Q1; Q3)	Median (Q1; Q3)	
Interval of symptom onset at admission	6.0 (3.7; 7.7)	7.0 (5.0; 8.0)	0.497 ^¥^
Length of stay	8.5 (5.0; 23.5)	8.0 (5.0; 14.0)	0.630 ^¥^

* Chi-Squared, ** Fisher’s Exat Test, *** T Test, ^¥^ Mann-Whitney Test. COPD, chronic obstructive pulmonary disease.

**Table 2:** Characteristics of COVID-19 patients undergoing brain imaging (CT/MRI).

#.	Sex	Age	Anosmia	Other Neurological Symptoms	Fever	Cough	Dyspnea	Oxygen Support	Brain Imaging	Thorax ct	Type of Care	Death
1	M	31	yes	seizure	yes	yes	yes	ventilator	normal (CT/MRI)	>50%	ICU	no
2	M	54	-	seizure	yes	yes	yes	O_2_ catheter	normal (CT)	<25%	urgency	no
3	F	72	no	headache	no	no	no	no	normal (CT)	<25%	urgency	no
4	M	46	-	headache	no	no	no	no	normal (CT)	<25%	internal	no
5	M	29	-	headache, seizure	yes	yes	no	no	cavernous sinus gaseous foci (CT)	<25%	urgency	no
6	M	60	-	dysesthesia, inferior limbs paresthesia	yes	yes	yes	ventilator	normal (CT)	>50%	internal	no
7	M	73	-	syncope	no	yes	no	no	hypodensity foci (CT)	25–50%	internal	no
8	F	84		-	no	no	no	no	intraparanchymal temporoparietal hematoma (CT)	25–50%	internal	yes
9	F	43	yes	headache	yes	yes	yes	no	normal (MRI)	25–50%	urgency	no
10	M	67	no	myopathy	yes	yes	yes	ventilator	normal (CT)	>50%	internal	no
11	F	53	-	headache	yes	yes	yes	no	normal (CT)	<25%	internal	No
12	F	69	-	seizure	yes	yes	yes	no	normal (CT)	<25%	internal	No
13	M	55	-	-	no	yes	no	no	normal (CT)	<25%	internal	no
14	M	81	-	alteration of consciousness	yes	yes	no	ventilator	normal (CT)	<25%	internal	yes
15	M	26		seizure, hemiparesis	no	no	no	no	temporo-occipital hypersignal on FLAIR, hemosiderosis (MRI)	>50%	ICU	no
16	F	34	yes	headache	yes	yes	no	no	olfactory bulb injury (MRI)	<25%	internal	no
17	F	43	no	headache, dizziness	no	yes	yes	no	olfactory bulb injury (MRI)	-	internal	no
18	M	40	yes	headache, hemiparesthesia, visual field fog	yes	yes	no	no	corpus callosum restricted DWI lesion and olfactory bulb injuries (MRI)	-	internal	no
19	F	35	yes	headache	yes	yes	no	no	olfactory bulb injury (MRI)	normal	internal	no
20	F	43	-	seizure	no	no	no	no	normal	<25%	internal	no
21	M	83	-	lowering of level of consciousness	yes	yes	no	O_2_ catheter	signs of past frontal and cerebellar injuries (MRI)	25–50%	ICU	no
22	M	62	-	severe depression, parkinsonism, coma	yes	no	yes	ventilator	olfactory bulb injury (MRI)	>50%	ICU	no
23	M	66	-	ischemic encephalopathy due to cardiac arrest	yes	yes	no	ventilator	normal (CT/MRI)	25–50%	ICU	no
24	F	25	yes	headache	yes	no	no	no	olfactory bulb injury (MRI)	normal	external	no
25	F	36	-	headache	-	-	-	no	olfactory bulb injury (MRI)	normal	external	no
26	M	71	-	Neurologic sequel	-	-	-	no	parietal subcortical microbleeding (MRI)	>50%	ICU	no
27	F	61	-	headache	-	-	-	no	cortical/subcortical hematomas and pale globes microbleedings + olfactory bulb injury (MRI)	>50%	internal	no
28	M	80	-	seizure, recurrent syncope	yes	yes	yes	O_2_ catheter	frontal microbleeding and olfactory bulb injury (MRI)	<25%	ICU	no
29	M	49	yes	seizure	yes	yes	yes	O_2_ catheter	normal (MRI)	<25%	ICU	no
30	F	70	-		yes	yes	yes	ventilator	microbleedings in base ganglia, brainstem and cerebellum (MRI)	>50%	ICU	yes
31	M	62	-	coma	yes	yes	yes	ventilator	cerebellum, corpus callosum and parietal white matterbleeding and restricted DWI small lesions (MRI)	>50%	ICU	no
32	M	40		left facial palsy	yes	No	no	no	geniculate ganglion injury and olfactory bulb injury (MRI)	-	external	no
33	M	41		encephalitis	-	-	-	-	olfactory bulb injury (MRI)	-	ICU	no
34	F	34		stroke and seizure	-	-	-	-	old ischemic lesion with restricted DWI areas/right middle cerebral artery stenosis/areas of hemorrhage and olfactory bulb injury (MRI)	<25%	external	no
35	M	82		desorientation	-	-	-	-	Small right parietal cortical gliosis (MRI)	>50%	internal	no

CT: computed tomography; MRI: magnetic radiology image; ICU: Intensive Care Unit; FST1: fat suppressed T1; FST1C post contrast fat suppressed T1; OB: olfactory bulb.

## Data Availability

All the relevant data on these cases has been published in the current article. No further data on these patients is available so as to preserve their anonymity, as well as, respecting the rules of medical and scientific ethics. *Study subjects or cohorts overlap*: Only 6 (six) studied subjects have overlapped with our previous studies (from 232 patients of the sample of this present study) one of them shown in Figure 6 F and another in Figure 10 B–D. These articles were cited on the figures described: (a) Five studied subjects (one shown again in Figure 6F of the present study) have been previously reported in our AJNR article: Aragão, M.d.F.V.V.; Leal, M.D.C.; Filho, O.Q.C.; Fonseca, T.M.; Valença, M.M. Anosmia in COVID-19 associated with injury to the olfactory bulbs evident on MRI. *Am. J. Neuroradiol.* 2020, *41*, 1703–1706, doi:10.3174/ajnr.A6675; (b) One of them (shown in Figure 10 B–D of the present study) was published at Aragão, M.F.V.V.; Leal, M.C.; Fonseca, T.M.; Cartaxo Filho, O.Q.; Valença, M.M. Reply. *Am. J. Neuroradiol.* 2020, doi:10.3174/ajnr.A6943. The previous version of this article with 876 cases of suspected Covid-19 was posted in the preprint server (MedRxIV) in January 2021 <https://www.medrxiv.org/content/10.1101/2020.12.28.20248957v1> on medRxiv (accessed on 29 March 2021). It was also published in the World Health Organization COVID-19 Global Literature on Coronavirus disease.
